# Correlation of Maternal Vitamin D Status in Early Pregnancy and Vitamin D Supplementation during Pregnancy with Atopic Dermatitis in Infants: A Prospective Birth Cohort Study

**DOI:** 10.3390/nu16132168

**Published:** 2024-07-08

**Authors:** Qianqian Zhang, Dongjian Yang, Qianwen Shen, Wei Li, Ruoxuan Li, Yanan Tang, Zhimin Lei, Baihe Li, Xiya Ding, Meng Ni, Ze Chen, Zhenying Lin, Chunyu Cheng, Dongting Yao, Yi Hu, Xiaorui Liu, Jiuru Zhao, Hao Chen, Zhiwei Liu

**Affiliations:** 1 International Peace Maternity and Child Health Hospital, School of Medicine, Shanghai Jiao Tong University, Shanghai 200030, China; qianqianzhang@shsmu.edu.cn (Q.Z.); djyang@shsmu.edu.cn (D.Y.); winnie_shen@sjtu.edu.cn (Q.S.); muzili158@sjtu.edu.cn (W.L.); lrx1228@sjtu.edu.cn (R.L.); yanan.tang@sjtu.edu.cn (Y.T.); leizhimin@sjtu.edu.cn (Z.L.); jylibaihe@sjtu.edu.cn (B.L.); sensa-ding@sjtu.edu.cn (X.D.); ni-m_zoe@sjtu.edu.cn (M.N.); chenze@sjtu.edu.cn (Z.C.); sjtu-linzhenying@sjtu.edu.cn (Z.L.); chengchunyu@sjtu.edu.cn (C.C.); yaodongting@sjtu.edu.cn (D.Y.); hnly5311050@sjtu.edu.cn (Y.H.); liuxiaorui@sjtu.edu.cn (X.L.); 730001668@shsmu.edu.cn (J.Z.); 2 Shanghai Key Laboratory of Embryo Original Diseases, Shanghai 200030, China; 3 Departments of Neonatology, Children’s Hospital of Shanghai, School of Medicine, Shanghai Jiao Tong University, Shanghai 200040, China

**Keywords:** vitamin D, atopic dermatitis, eczema, assisted reproductive technology, vitamin D supplementation, exclusive breastfeeding

## Abstract

Objective: This study aimed to investigate the association of maternal first-trimester vitamin D levels and vitamin D supplementation during pregnancy with infant atopic dermatitis (AD) and to determine the effect of variables such as mode of conception on the association. Methods: This study was based on the Shanghai sub-cohort of the International Birth Cohort of China. A total of 4051 woman–infant pairs with singleton pregnancies were recruited. Vitamin D deficiency and insufficiency were defined as serum 25-hydroxyvitamin D concentrations of 25 and 50 nmol/L, respectively. AD in infants was assessed during the first six months using a standardized questionnaire based on the British Working Party criteria. Modified Poisson regression estimated the association between maternal vitamin D status and infant AD. Results: The risk of AD in infants was higher in women with deficient 25-hydroxyvitamin D levels in the first trimester (RR: 1.77, 95% CI: 1.41–2.23). This increased risk was seen in naturally conceived pregnancies, but not in those conceived using assisted reproductive technology (ART). The incidence of AD decreased in infants of mothers who took multi-vitamin (RR: 0.79, 95% CI: 0.67–1.98) and vitamin D supplements (RR: 0.51, 95% CI: 0.37–0.71) compared to those whose mothers did not take any supplements. Maternal vitamin D deficiency had varying effects on AD risk based on passive smoking exposure and breastfeeding patterns. Conclusions: Our findings highlight the importance of monitoring and supplementing vitamin D during pregnancy, especially in specific maternal populations, to reduce the risk of AD in offspring.

## 1. Introduction

Atopic dermatitis (AD), also known as atopic eczema, is a chronic inflammatory skin condition associated with epithelial, immune, and environmental factors and characterized by redness, swelling, scaling, oozing, and dryness. It affects up to 20% of the pediatric population, with a growing prevalence. AD typically develops in infancy, often preceding other allergic manifestations, such as asthma and allergic rhinitis. This sequential development of allergic conditions is often referred to as the atopic march or atopic triad [1]. More importantly, individuals with AD are more likely to develop asthma and allergic rhinitis [2,3].

The factors contributing to AD development include deficient epidermal barrier, disruption of the skin’s microbial balance, and altered immune response. While the main drivers of AD remain subject to ongoing debate, growing evidence indicates a close interaction between the skin barrier biology and immune mechanisms [4]. AD partly originates in utero, with environmental factors and genetic susceptibility influencing immune system development and altering the skin barrier function [5]. Understanding the role of early-life environmental exposures, such as the maternal nutritional status, are crucial for AD prevention in utero and for reducing the risk of AD in infants [6,7].

Vitamin D deficiency is highly prevalent among pregnant women. We previously identified that a significant proportion of pregnant women in the Shanghai region experienced vitamin D insufficiency during the first trimester, with rates as high as 70% [8]. The American Institute of Medicine (IOM) recommends that serum 25(OH)D levels above 50 nM are considered sufficient for vitamin D to prevent disorders of bone development and calcium homeostasis. However, pregnancy or offspring outcomes have no recommended reference range. Given the crucial role of vitamin D in immune regulation, numerous studies have demonstrated that the maternal vitamin D status during pregnancy is associated with the risk of AD and other allergic diseases in infants; however, evidence on the protective effects of vitamin D is inconsistent [9,10,11,12,13]. It has been reported that vitamin D interacts with the skin barrier-related factor, filaggrin, affecting sensitization load in non-Black participants and regulating the skin allergy [14]. Additionally, factors such as assisted reproductive technology (ART), lifestyle changes, and environmental pollution may further influence maternal vitamin D levels and AD occurrence.

The aim of this prospective cohort study was to investigate the association between maternal first-trimester vitamin D levels, including vitamin D supplementation during pregnancy, and the onset of infant AD within the first six months of life. It also considered whether ART conception, vitamin D supplementation, breastfeeding patterns, and other factors impact maternal vitamin D levels and infant AD risk to inform prevention strategies for AD and related allergic diseases.

## 2. Methods

### 2.1. Study Population

**Figure 1:**
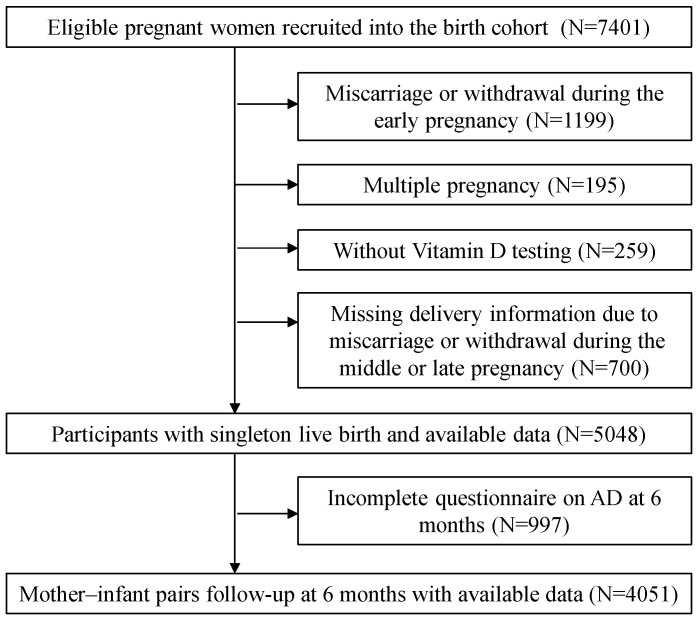
Flowchart of this study.

The study population comprised the Shanghai sub-cohort of the International Birth Cohort of China, recruited from October 2017 to September 2021 at the International Peace Maternity and Child Health (IPMCH) Hospital, affiliated with the Shanghai Jiao Tong University School of Medicine. The cohort recruited women who intended to undergo ART before pregnancy, and conceived naturally in early pregnancy. As illustrated in the flowchart (Figure 1), 7401 pregnant women were initially enrolled in this study. Among these, we excluded 1199 women who had a miscarriage or withdrew voluntarily during early pregnancy, 195 women who had multiple pregnancies, 259 women who did not undergo vitamin D testing, 700 women who were missing delivery information owing to miscarriage or withdrawal during late pregnancy, and 997 mother–infant pairs who did not complete the questionnaire on AD at 6 months. Ultimately, 4051 mother–infant pairs were included in the final analysis. Written informed consent was obtained from all participants after full disclosure, and this study was approved by the Medical Research Ethics Committee of IPMCH (approval number: GKLW2016-21). This study adhered to the Strengthening the Reporting of Observational Studies in Epidemiology (STROBE) guidelines.

### 2.2. Outcomes

The outcomes were based on the assessment of AD at 6 months, conducted by trained professionals using a standardized questionnaire based on the British Working Party diagnostic criteria [15]. The diagnostic criteria include a history of itchy skin and at least two of the following: (a) a history of skin involvement in areas such as the elbow, back of the knee, front of the ankle, cheek, or neck; (b) a history of generalized skin dryness; and (c) visible eczema in skin folds or on the cheeks, forehead, and limbs. 

### 2.3. Vitamin D Detection

Serum first-trimester 25(OH)D concentrations were used to assess the maternal vitamin D status. Maternal blood samples (5 mL) were collected from the median cubital vein at antenatal visits during early pregnancy (9–13 weeks), and serum was extracted within 2 h of collection. Quantitative analysis of 25(OH)D was performed using the chemiluminescent particle immunoassay in an Architect I2000SR autoanalyzer (Abbott Diagnostics, Chicago, IL, USA). The detection range of the analyzer was 2.00–400.00 nmol/L. Owing to the lack of consensus regarding the reference standard for serum 25(OH)D concentration during pregnancy, this study defined serum 25(OH)D levels as follows: >50.0 nmol/L as vitamin D sufficient, 25.1–50 nmol/L as insufficient, and <25 nmol/L as deficient, according to the research hypotheses and previous findings [16].

### 2.4. Covariates

This was a prospective hospital-based birth cohort study, and variables were obtained through questionnaires and clinical records. Maternal data, such as gestational age, pre-pregnancy body mass index (BMI), mode of conception, ethnicity, education level, family income, vitamin D supplementation, exercise during pregnancy, passive smoking, as well as the sex and breastfeeding pattern of the infant were collected through questionnaires. Additionally, maternal parity, gravidity, medical history (diabetes, hypertension, thyroid disease), and infant information, such as the gestational age at birth, birth weight, mode of delivery, and season of birth, were obtained from clinical records. Pre-pregnancy BMI was calculated based on pre-pregnancy weight and height, which were classified as <18.5, 18.5–23.9, and ≥24 kg/m^2^, according to the classification of the Chinese population [17]. The family’s annual income was classified as ≤300,000 and >300,000 RMB. The breastfeeding patterns of infants from birth to 6 months were investigated and categorized into exclusive and non-exclusive breastfeeding (including artificial formula feeding and mixed feeding). Recognizing the seasonal influence on AD and vitamin D levels, the birth months were classified into spring (March, April, and May), summer (June, July, and August), autumn (September, October, and November), and winter (December, January, and February). The mode of conception was categorized as ART and natural conception. Information on vitamin D supplementation during early, middle, and late pregnancy was categorized as follows: none, only multi-vitamin supplements, and vitamin D supplements (supplementation with individual vitamin D alone or in addition to a multi-vitamin supplement). This information was collected using a questionnaire based on the types of supplements used. Examples of multi-vitamin supplements include Elevit, Materna, KonNaD, and Centrum. Examples of vitamin D supplements include Swisse, Zymafluor, and D.Cal. 

### 2.5. Statistical Analysis

Means [standard deviation (SD)] and frequencies (percentage) were used, respectively, to summarize continuous and categorical variables of the study population.

To estimate the association between vitamin D status in the first trimester and infant AD, we modified the Poisson regression [18], adjusting for confounders such as gestational age, pre-pregnancy BMI, ethnicity, educational level, history of allergy, drinking, smoking, parity, gravidity, birth season, mode of delivery, infant sex, and infant feeding pattern from birth to 6 months of age. The serum 25(OH)D level was modeled as a nominal variable based on grouping criteria, with ≥50 nmol/L as the reference group. We utilized modified Poisson regression for robust (sandwich) variance estimation of binary [18] and non-overlapping outcomes rather than logistic regression to estimate the relative risk (RR) of violating the rare disease assumption. We used robust (sandwich) variance to estimate 95% confidence intervals (95% CIs) and *p*-values because the mean–variance correlation of the Poisson distribution may not be appropriate for our binary outcomes. We tested the confounders for each model’s outcome, and the covariates were selected or excluded based on confounding plausibility, changes in the effect estimate of the variable of interest, and the reduction of the model’s residual variance. Model 1 was adjusted for maternal gestational age, pre-pregnancy BMI, mode of conception, maternal ethnicity, gravidity, parity, maternal education level, use of alcohol during pregnancy, smoking during pregnancy, exercise during pregnancy, passive smoking during pregnancy, family income, season of birth of the infant, and parental allergy history. Model 2 included adjustments from Model 1 plus for hypertension during pregnancy, diabetes mellitus during pregnancy, and thyroid disease during pregnancy. Model 3 was further adjusted for vitamin D supplementation during early pregnancy.

Continuously distributed serum 25(OH)D levels were used to estimate the nonlinear effects by fitting a restricted cubic spline (RCS) function after adjusting for the confounders in Model 3. Knots were set to the 10th, 50th, and 90th percentiles. The variables were classified as dichotomous according to whether 25(OH)D was deficient (<25 nmol/L) or not (≥25 nmol/L). Following this, subgroup analyses were conducted according to the following factors and presented in forest plots: gestational age (<35, ≥35, years), pre-pregnancy BMI (<18.5, 18.5–23.9, ≥24 kg/m^2^), annual family income (≤300,000 and >300,000 RMB), infant’s sex (male, female), mode of conception (ART, natural conception), birth season (spring, summer, autumn, winter), breastfeeding pattern (exclusive breastfeeding, non-exclusive breastfeeding), parity (1, ≥2), mode of delivery (vaginal delivery, cesarean section), passive smoking during pregnancy (no, yes), parental history of allergies (no, yes), vitamin D supplementation during pregnancy (no, yes).

To enhance the robustness of our results, we conducted a sensitivity analysis using propensity score inverse probability weighting (PS-IPTW) to balance potential confounders and improve the robustness of the results. Propensity scores (PSs) were calculated for participants, divided into groups based on vitamin D levels (categorized as deficiency, insufficiency, or sufficiency) in the first trimester, combined with the distribution of potential confounders. These scores were then used in multifactor-weighted Poisson regression with robust (sandwich) variance to estimate associations between vitamin D status and infant AD [18]. 

All analyses and presentation of results in this study were analyzed with R (version: 4.03), and *p* < 0.05 (2-sided) was considered statistically significant.

## 3. Results

### 3.1. Characteristics of the Study Population

A total of 4051 mother–infant pairs that fulfilled the inclusion criteria were enrolled in this study (Table 1). Among them, 748 (18.5%) conceived via ART. The distribution of maternal 25(OH)D concentrations in the first trimester was as follows: 581 (14.3%) mothers had deficient levels, 1790 (44.2%) had insufficient levels, and 1680 (41.5%) had sufficient levels. During the first, second and third trimesters of pregnancy, respectively, 183 (4.5%), 437 (10.8%), and 505 (12.5%) mothers reported supplementing with individual vitamin D alone or in addition to a multi-vitamin regimen. However, supplementation with only multi-vitamin was reported by 2481 (61.2%), 2640 (65.2%), and 2328 (57.5%) mothers. Among the 4051 infants, 2038 (50.3%) were male, and 2196 (54.2%) were delivered vaginally. Additionally, 1112 (27.5%) had a parental history of allergies, and 1388 (35.9%) were exclusively breastfed for 6 months. The prevalence of AD during the first six months of life was 13.7%. 

### 3.2. Association of Maternal Serum 25(OH)D Status with Infant AD

**Figure 2:**
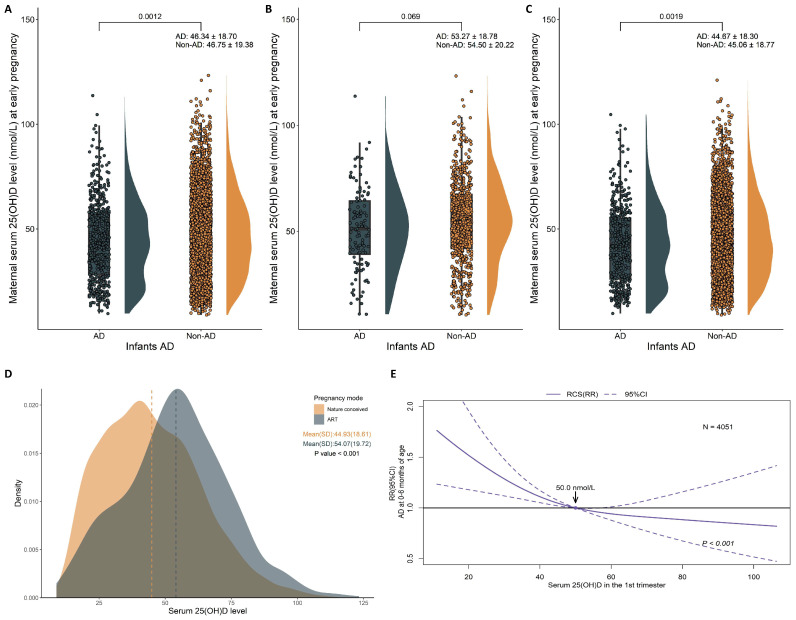
Distribution of maternal serum 25(OH)D levels between infants with AD and those without (non-AD) in the overall population (**A**), mothers who conceived through ART (**B**), and mothers who conceived naturally (**C**). The association between maternal first-trimester vitamin D levels and the AD in infants. Comparison of maternal serum 25(OH)D concentrations between mothers who conceived naturally and those who conceived through ART (**D**). The dashed lines indicate the standard deviation (SD) values of each population. The non-linear association between maternal first-trimester vitamin D levels and the risk of AD in infants (**E**). Plots show the non-linear regression models for maternal first-trimester serum 25(OH)D concentrations and RR of infant AD within 6 months of age (solid line) with 95% CI (dashed line,) assessed using restricted cubic spline (RCS). Knots were set to the 10th, 50th, and 90th percentiles. The analysis was adjusted for all covariates from adjusted Model 1, which included maternal gestational age, pre-pregnancy BMI, mode of conception, maternal ethnicity, gravidity, parity, maternal education level, drinking during pregnancy, smoking during pregnancy, exercise during pregnancy, passive smoking during pregnancy, family income, season of birth of infant, parental allergy history, hypertension during pregnancy, diabetes mellitus during pregnancy, and thyroid disease during pregnancy.

Infants who developed AD within 6 months had significantly lower mean maternal serum 25(OH)D concentrations in the first trimester (*p* = 0.0012), and the incidence of vitamin D deficiency was higher in mothers whose infants had AD (Figure 2A). For the ART-conceived cases (Figure 2B), no significant difference in mean maternal serum 25(OH)D concentrations was observed between infants with and without AD (*p* = 0.069). In naturally conceived cases (Figure 2C), the mean serum 25(OH)D concentrations were significantly lower in mothers of infants with AD (*p* = 0.0019). In addition, as shown in Figure 2D, compared to women who conceived naturally, the mean of maternal serum 25(OH)D concentrations in the first trimester were significantly higher in mothers who conceived via ART (44.93 vs. 54.07 nmol/L, *p* < 0.001). The non-linear association between maternal first-trimester 25(OH)D levels and the risk of AD in offspring from birth to 6 months is shown in Figure 2E. Pregnant women with serum 25(OH)D levels below 50 nmol/L, which is considered a sufficient reference value, exhibited an elevated risk of AD in their infant as the serum 25(OH)D concentration decreased (*p* = 0.0448). 

As shown in Table 2, compared to the mothers with sufficient 25(OH)D levels, those with deficient levels had a 53% higher risk of infants developing AD within 6 months of age (RR: 1.53, 95% CI: 1.24–1.88). This effect remained significant after adjusting for the confounders in Model 1 (RR: 1.48, 95% CI: 1.20–1.83) and Model 2 (RR: 1.49, 95% CI: 1.20–1.84). In Model 3, with an additional adjustment for vitamin D supplementation, the risk of AD increased further (RR: 1.77, 95% CI: 1.41–2.23). Insufficient maternal 25(OH)D levels were not associated with the risk of AD in infants.

The IPTW analysis showed that the risk effect of maternal first-trimester vitamin D deficiency on infants with AD was sustained. Compared to mothers with sufficient vitamin D levels, mothers with deficient vitamin D levels in early pregnancy had infants with a 154% increased risk of AD during the first 6 months of life (RR: 1.54, 95% CI: 1.22–1.93). The risk effect of maternal vitamin D deficiency in the first trimester still existed after adjusting for the confounding factors in Model 1 (RR: 1.54, 95% CI: 1.22–1.89), Model 2 (RR: 1.52, 95% CI: 1.22–1.89), and Model 3 (RR: 1.79, 95% CI: 1.42–2.27).

### 3.3. Association of Maternal Vitamin D Supplementation with Infant AD

As shown in Table 3, the incidence of AD in infants during the first 6 months was significantly decreased in pregnant women who took multi-vitamin supplements (Model 1, RR: 0.80, 95% CI: 0.68–0.94; Model 2, RR: 0.79, 95% CI: 0.67–1.98) or vitamin D supplements (Model 1, RR: 0.52, 95% CI: 0.37–0.72; Model 2, RR: 0.51, 95% CI: 0.37–0.71), compared to those who did not take any vitamin D supplements in the third trimester. However, the pregnant women who took only multi-vitamin supplements or vitamin D supplements in the second trimester were not associated with the risk of AD in infants.

As shown in Table 4, among pregnant women with insufficient vitamin D levels (serum 25(OH)D concentrations < 50.0 nmol/L) in the first trimester, those who took only multi-vitamin supplements and those who took vitamin D supplements had a decreased incidence of AD in their infants (RR: 0.72, 95% CI: 0.53–0.97 and RR: 0.44, 95% CI: 0.26–0.74), compared to pregnant women who did not take any vitamin D supplements during the second trimester. The protective effect of multi-vitamin supplements slightly increased after adjusting for the confounding factors in both Model 1 (RR: 0.66, 95% CI: 0.48–0.92) and Model 2 (RR: 0.64, 95% CI: 0.46–0.89). Similarly, the protective effect of vitamin D supplements also showed a slight increase after adjustment in Model 1 (RR: 0.40, 95% CI: 0.23–0.71) and Model 2 (RR: 0.38, 95% CI: 0.22–0.68).

Compared to the infants of pregnant women who did not take any vitamin D supplements in the third trimester, the incidence of AD in infants of pregnant women who took vitamin D supplements significantly decreased by 46% (RR: 0.54, 95% CI: 0.34–0.88). The protective effect of vitamin D supplements in the third trimester slightly increased after adjusting for the confounding factors in Model 1 (RR: 0.40, 95% CI: 0.23–0.70) and Model 2 (RR: 0.41, 95% CI: 0.23–0.71). 

Among the pregnant women with sufficient vitamin D levels (serum 25(OH)D concentrations ≥ 50.0 nmol/L) in the first trimester, those taking vitamin D supplements had a lower prevalence of infant AD (RR: 0.65, 95% CI: 0.44–0.96) compared to those who did not take vitamin D supplements in the third trimester. 

The risk of AD was decreased by 37% (RR: 0.63, 95% CI: 0.42–0.94) and 38% (RR: 0.62, 95% CI: 0.41–0.94) after adjusting for the confounding factors in Models 1 and 2, respectively. Furthermore, compared to the infants of pregnant women who did not take any type of vitamin D supplements in the third trimester, the incidence of AD in the infants of pregnant women who took multi-vitamin supplements decreased by 20% (RR: 0.80, 95% CI: 0.65–0.98) after adjusting for the confounders in Model 2.

As shown in Supplementary Table S1, regardless of the type of vitamin D supplementation, the protective effect of taking continuous vitamin D supplements during both the second and third trimesters on infant AD remained significant after adjusting for the confounding factors in Model 1 (RR: 0.72, 95% CI: 0.59–0.88) and Model 2 (RR: 0.69, 95% CI: 0.56–0.85). Among the subset of pregnant women with insufficient vitamin D levels (serum 25(OH)D concentrations < 50.0 nmol/L) in the first trimester, supplementation with any type of vitamin D during both the second and third trimesters decreased the risk of infant AD after adjusting for the confounding factors in both Model 1 (RR: 0.58, 95% CI: 0.44–0.84) and Model 2 (RR: 0.57, 95% CI: 0.39–0.84).

### 3.4. Subgroup Analysis of Variables Influencing the Interaction between Maternal Vitamin D Deficiency and Offspring AD

**Figure 3:**
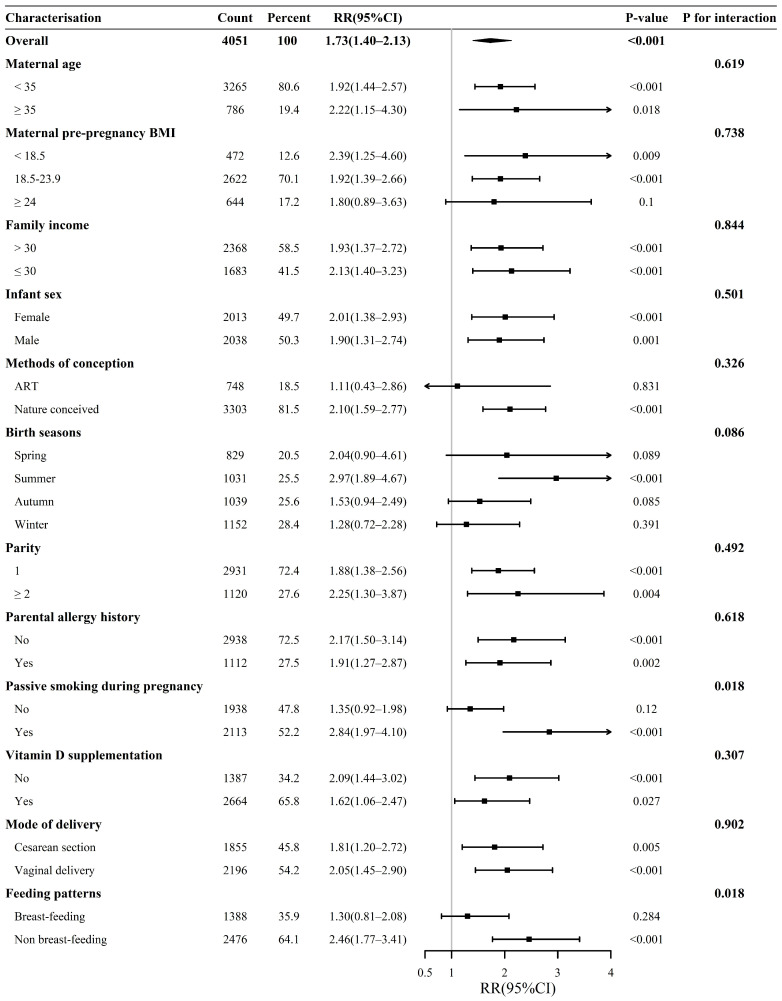
Subgroup analysis of maternal first-trimester vitamin D levels and the risk of AD in infants. The subgroup analyses were conducted according to the variable factors and presented in forest plots, categorized by whether maternal first-trimester serum 25(OH)D was deficient (<25 nmol/L) or not (≥25 nmol/L). The analyses were adjusted for all covariates from the adjusted Model 1, which included maternal gestational age, pre-pregnancy BMI, mode of conception, maternal ethnicity, gravidity, parity, maternal education level, drinking during pregnancy, smoking during pregnancy, exercise during pregnancy, passive smoking during pregnancy, family income, season of birth of infant, parental allergy history, hypertension during pregnancy, diabetes mellitus during pregnancy and thyroid disease during pregnancy. Stratification variables were not included in the adjustment.

We conducted a subgroup analysis and estimated the *p* values for the interaction terms to explore the association between maternal vitamin D status in the first trimester and infant AD, considering multiple variables. Overall (Figure 3), compared to the mothers with sufficient and insufficient vitamin D levels (serum 25(OH)D concentrations ≥ 25.0 nmol/L) in the first trimester, those with deficient vitamin D levels (serum 25(OH)D concentrations < 25.0 nmol/L) had infants with an increased risk of AD during the first 6 months of life (RR: 1.73, 95% CI: 1.40–2.13). When stratified by the maternal pre-pregnancy BMI, the risk effect of maternal vitamin D deficiency in the first trimester on infant AD during the first 6 months of life remained in the subgroup of underweight (RR: 2.39, 95% CI: 1.25–4.60) and normal weight (RR: 1.92, 95% CI: 1.39–2.66) mothers, but not in the subgroup of overweight or obese (OWO) mothers. An increased risk of AD was also observed in the infants who were naturally conceived (RR: 2.10, 95% CI: 1.59–2.77), but not in those who were conceived through ART. However, the mode of conception did not influence the interaction between maternal vitamin D deficiency and the risk of AD in infants. When stratified by the birth seasons of the offspring, maternal vitamin D deficiency in the first trimester was only associated with AD within 6 months of age in the infants born in summer (RR: 2.97, 95% CI: 1.89–4.67). Breastfeeding patterns were found to interact with the association between maternal vitamin D deficiency and the risk of infants developing AD (*p* for interaction = 0.018). Among non-exclusively breastfed infants within 6 months of age, the mothers with deficient vitamin D levels in the first trimester had infants with a 146% increased risk of AD (RR: 2.46, 95% CI: 1.77–3.41) compared to mothers with sufficient and insufficient vitamin D. However, this relationship did not exist among exclusively breastfed infants. In addition, there was a significant interaction effect of passive smoking during pregnancy on the relationship between maternal vitamin D deficiency and AD in infants (*p* for interaction = 0.018). For pregnant women exposed to passive smoking during pregnancy, the infants of those with deficient vitamin D levels in the first trimester had a 184% increased risk of AD (RR: 2.84, 95% CI: 1.97–4.10) compared to the infants born to mothers with sufficient or insufficient vitamin D. However, this correlation did not exist among those not exposed to passive smoking during pregnancy. Furthermore, pregnant women who took vitamin D supplements appeared to have infants at a lower risk of developing AD owing to first-trimester vitamin D deficiency, compared to pregnant women who did not take any type of vitamin D supplements (RR: 1.62, 95% CI: 1.06–2.47 vs. RR: 2.09, 95% CI: 1.44–3.02).

In the cases conceived through ART (Supplementary Figure S1A), maternal vitamin D deficiency levels did not increase the risk of infants developing AD during the first 6 months of life, and the stratified analysis did not show an increased risk of AD occurrence. The infants who were conceived naturally (Supplementary Figure S1B) by mothers with deficient vitamin D levels in the first trimester had an 83% increased risk of AD during the first 6 months of life (RR: 1.83, 95% CI: 1.47–2.28), compared to their counterparts born to mothers with sufficient or insufficient vitamin D levels. The risk of infant AD was similar to that observed in the entire study population when stratified by maternal pre-pregnancy BMI, family income, infant sex, birth seasons, feeding patterns, parity, mode of delivery, passive smoking status during pregnancy, parental allergy history, and vitamin D supplementation during pregnancy.

## 4. Discussion

### 4.1. Main Findings

Over 50% of women were found to have vitamin D insufficiency in the first trimester, and more than 10% of infants developed AD within the first 6 months. Maternal first-trimester vitamin D deficiency is associated with an increased risk of offspring AD within the first 6 months. Factors such as the mode of conception, maternal pre-pregnancy BMI, breastfeeding patterns, and passive smoking during pregnancy influenced this association. Additionally, vitamin D or multi-vitamin supplementation in late pregnancy was linked to a reduced risk of infant AD. The protective effect of individual Vitamin D supplements on AD was greater than that of multi-vitamin supplements containing vitamin D.

### 4.2. Interpretation

Previously, several studies have explored the association between maternal vitamin D and the risk of AD in infants [9,11,12,19], as well as the effect of antenatal vitamin D supplementation on AD [10]. Most studies support the claim that maternal circulating and fetal cord blood levels of vitamin D are inversely associated with the risk of AD; however, the conclusions regarding the effect of antenatal vitamin D supplementation on the risk of AD in infants remain inconsistent [20]. In this study, we confirmed that maternal vitamin D exerts a protective effect against the occurrence of AD in infants within 6 months of age. Furthermore, based on the data collected in the questionnaire survey, supplementation with vitamin D or multiple vitamins in late pregnancy is associated with a decreased risk of AD in infants within 6 months of age. Notably, our findings show that if a mother has insufficient vitamin D levels in early pregnancy, supplementation with vitamin D or multi-vitamin in mid-pregnancy could also reduce the risk of AD in infants in the first six months of life. Furthermore, the effect of maternal supplementation with multiple vitamins and vitamin D in late pregnancy on reducing the risk of AD in infants is more pronounced in mothers with insufficient vitamin D levels compared to those with sufficient vitamin D levels. These results indicate the necessity of multiple vitamins and individual vitamin D supplementation in the second and third trimesters of pregnancy to reduce the risk of AD in infants.

Our study also included a population of women who conceived through ART. Women with higher serum vitamin D levels have a greater chance of successful conception through ART [21]. However, the association between maternal vitamin D levels and the risk of AD in infants who were conceived through ART has rarely been reported. In this study, the vitamin D levels in the first trimester of mothers who conceived through ART were significantly higher than in mothers who conceived naturally. The incidence of AD in infants within 6 months of age in those conceived through ART was higher than that in those conceived naturally, but the difference was not significant. In cases conceived through ART, no protective effect of maternal vitamin D levels or vitamin D supplementation during pregnancy was observed on offspring with AD within 6 months of age. However, such a protective effect exists in cases conceived naturally. Owing to the differing levels of various hormones in mothers who conceived through ART compared to those who conceived naturally [22,23], there may be variations in the vitamin D levels, the incidence of AD in offspring within the first 6 months of age, and the association between maternal vitamin D levels and AD in infants.

Our findings also revealed various pre-pregnancy and pregnancy factors, including pre-pregnancy BMI, breastfeeding patterns, and maternal exposure to passive smoking during pregnancy, which influence the association between maternal first-trimester vitamin D and the risk of AD in infants. Maternal obesity before pregnancy has been shown to predict poor vitamin D status during pregnancy [24]. Additionally, there is a negative correlation between pre-pregnancy BMI and maternal vitamin D levels in the first trimester [25]. In our study, maternal vitamin D levels did not affect the risk of infants developing AD within the first 6 months of age among mothers who were OWO before pregnancy. However, in mothers who were underweight or with normal weight, maternal vitamin D deficiency increased the risk of AD in their infants. Thus, owing to the lower serum vitamin D levels in individuals who are OWO, the protective effect of maternal vitamin D on infants AD could not be accurately assessed at a low concentration range. Our findings suggest the importance of identifying and treating low vitamin D levels in mothers who are not OWO before pregnancy to reduce the risk of AD in infants. 

It is undeniable that combining maternal interventions and breastfeeding can lower the risk of food allergies in children [26]. Furthermore, vitamin D levels in breast milk are inversely associated with the severity of AD in exclusively breastfed infants within the first six months [27]. In this study, maternal vitamin D deficiency was not associated with an increased risk of AD in exclusively breastfed infants and it was found to be a risk factor in non-exclusively breastfed infants. However, the effect of breastfeeding on AD remains controversial [28]. A systematic review and meta-analysis of prospective cohort study found breastfeeding was associated with a decreased risk of AD when the analysis was restricted to the studies comparing breastfeeding with conventional formula feeding [29], and another study found weak evidence for a protective effect of breastfeeding against AD in cohorts with atopic heredity [30]. Thus, components of breast milk, such as polyunsaturated fatty acids and arachidonic acid [31,32], may attenuate maternal vitamin D deficiency on the risk of AD in infants and the allergens in formula milk [33] could exacerbate the risk effect.

Maternal vitamin D deficiency was associated with an increased risk of AD in infants whose mothers were exposed to passive smoking during pregnancy in this study; however, it was not a risk factor in mothers who were not exposed to passive smoking during pregnancy. Previous systematic review and meta-analysis studies suggested that passive smoking during pregnancy is associated with an increased risk of developing infant AD, whereas active smoking during pregnancy is not [34]. Thus, we deduced that passive smoking during pregnancy exacerbates the effect of maternal vitamin D deficiency on the development of AD by altering the infants’ immune response [35] and other mechanisms.

The vitamin D receptor (VDR) agonists can induce the Tregs population and the gene expression of barrier and antimicrobial functions in the allergen-triggered AD-like skin of mice [36]. It has been reported that prenatal vitamin D supplementation can attenuate the development of airway hyperresponsiveness in offspring with allergen-triggered asthma and reduce the numbers of Th2/Th17 cells [37]. However, the underlining mechanism of maternal vitamin D associated with AD in offspring is still not clear, and whether maternal vitamin D affects the immune development and skin barrier function of offspring needs further clarification. 

### 4.3. Strengths and Limitations

In this prospective mother–child cohort study, we recruited participants for 4 years and followed 4051 mother–infant pairs until the infants reached 6 months of age. The main strengths of our study lie in its large population and low rate of loss to follow-up, as well as its inclusion of cases conceived both naturally and through ART. However, the outcome (AD in infants) was based on a questionnaire survey that did not include the severity scoring index of AD and only recorded the presence or absence of AD. Additionally, the pre-pregnancy BMI, vitamin D supplementation during pregnancy, breastfeeding patterns, and offspring AD outcomes were based on self-reported data, which may have been subject to reporting bias. Moreover, the dose of maternal daily vitamin D supplementation could not be determined.

## 5. Conclusions

Maternal serum 25(OH)D deficiency in the first trimester is linked to an increased risk of offspring AD within the first 6 months of life. Vitamin D or multiple vitamin supplementation during middle and late pregnancy reduces this risk, but only in cases conceived naturally and not through ART. Pre-pregnancy BMI, breastfeeding patterns, and exposure to passive smoking during pregnancy also influenced this association. Our findings emphasize the importance of monitoring and supplementing vitamin D during pregnancy, to reduce the risk of AD in offspring. This study provides a theoretical basis for the importance of vitamin D level management during pregnancy. 

## Figures and Tables

**Table 1:** Demographic characteristics of the study population (N = 4051).

Characteristics	Summary
Maternal characteristics	
Age, mean (SD), years	31.5 (3.9)
Pre-pregnancy BMI, mean (SD), kg/m^2^	21.6 (4.8)
Ethnical, *n* (%)	
Han	3970 (98.0)
Others	81 (2.0)
Parity, *n* (%)	
1	2931 (72.4)
≥2	1120 (27.6)
Gravidity, *n* (%)	
1	1959 (48.4)
2	116 2(28.7)
≥3	929 (22.9)
Educational level, *n* (%)	
Below bachelor’s degree	993 (24.5)
Bachelor’s degree	2157 (53.2)
Master or above	901 (22.2)
Family income, *n* (%)	
≤20	1311 (32.4)
20–30	372 (9.2)
>31	2368 (58.5)
Pregnancy mode, *n* (%)	
ART	748 (18.5)
Nature of conception	3303 (81.5)
Drinking status during pregnancy, *n* (%)	
No	2085 (52.0)
Yes, but quit	1823 (45.4)
Yes, still	105 (2.6)
Smoking status during pregnancy, *n* (%)	
No	3935 (97.1)
Yes, but quit	100 (2.5)
Yes, still	16 (0.4)
Serum vitamin D status, *n* (%)	
≤25	581 (14.3)
25.1~50	1790 (44.2)
>50	1680 (41.5)
Vitamin D supplementation in early pregnancy, *n* (%)	
None	1387 (34.2)
Only multi-vitamin supplements	2481 (61.2)
Vitamin D supplements	183 (4.5)
Vitamin D supplementation in mid-pregnancy, *n* (%)	
None	974 (24.0)
Only multi-vitamin supplements	2640 (65.2)
Vitamin D supplements	437 (10.8)
Vitamin D supplementation in late pregnancy, n (%)	
None	1218 (30.1)
Only multi-vitamin supplements	2328 (57.5)
Vitamin D supplements	505 (12.5)
Infant characteristics	
Birth season, *n* (%)	
Spring	829 (20.5)
Summer	1031 (25.5)
Autumn	1039 (25.6)
Winter	1152 (28.4)
Infant sex, *n* (%)	
Female	2013 (49.7)
Male	2038 (50.3)
Delivery modes, *n* (%)	
Cesarean section	1855 (45.8)
Vaginal delivery	2196 (54.2)
Parental allergy history, n (%)	
No	2938 (72.5)
Yes	1112 (27.5)
Breastfeeding patterns, n (%)	
Artificial formula feeding	151 (3.9)
Exclusive breastfeeding	1388 (35.9)
Mixed feeding	2325 (60.1)
AD 0–6 months of age, n (%)	
No	3494 (86.3)
Yes	557 (13.7)

**Table 2:** Association of maternal vitamin D status in the first trimester with offspring AD within the first 6 months of age (N = 4051).

Serum 25(OH)D Level (nmol/L)	Unadjusted Model	Adjusted Model 1 ^a^	Adjusted Model 2 ^b^	Adjusted Model 3 ^c^
	RR (95%CI)	RR (95%CI)	RR (95%CI)	RR (95%CI)
>50	Ref.	Ref.	Ref.	Ref.
25.1~50	1.04 (0.88,1.24)	1.03 (0.86,1.23)	1.03 (0.86,1.23)	1.11 (0.93,1.34)
≤25	1.53 (1.24,1.88) **	1.48 (1.20,1.83) **	1.49 (1.20,1.84) **	1.77 (1.41,2.23) **
Sensitivity analysis by PS-IPTW				
>50	Ref.	Ref.	Ref.	Ref.
25.1~50	1.03 (0.86,1.25)	1.04 (0.87,1.25)	1.05 (0.87,1.25)	1.12 (0.93,1.35)
≤25	1.54 (1.22,1.93) **	1.52 (1.22,1.89) **	1.52 (1.22,1.89) **	1.79 (1.42,2.27) **

**: <0.001. ^a^ Adjusted for maternal gestational age, pre-pregnancy BMI, mode of conception, maternal ethnicity, gravidity, parity, maternal education level, drinking during pregnancy, smoking during pregnancy, exercise during pregnancy, passive smoking during pregnancy, family income, season of birth of infant, parental allergy history. ^b^ Adjusted for all covariates from adjusted Model 1 and additionally adjusted for hypertension during pregnancy, diabetes mellitus during pregnancy, and thyroid disease during pregnancy. ^c^ Adjusted for all covariates from adjusted Model 2 and additionally adjusted for vitamin D supplementation during early pregnancy.

**Table 3:** Association of vitamin D supplementation during pregnancy with infant AD within the first 6 months of age (N = 4051).

Vitamin D Supplementation	Unadjusted Model	Adjusted Model 1 ^a^	Adjusted Model 2 ^b^
	RR (95%CI)	RR (95%CI)	RR (95%CI)
During early pregnancy			
None	Ref.	Ref.	Ref.
Only multi-vitamin supplements	1.15 (0.97,1.37)	1.13 (0.95,1.34)	1.13 (0.95,1.34)
Vitamin D supplements	1.32 (0.93,1.89)	1.34 (0.95,1.90)	1.35 (0.96,1.91)
During mid-pregnancy			
None	Ref.	Ref.	Ref.
Only multi-vitamin supplements	0.90 (0.75,1.07)	0.84 (0.70,1.01)	0.81 (0.67,0.97) *
Vitamin D supplements	0.80 (0.60,1.08)	0.78 (0.58,1.06)	0.75 (0.55,1.01)
During late pregnancy			
None	Ref.	Ref.	Ref.
Only multi-vitamin supplements	0.87 (0.74,1.03)	0.80 (0.68,0.94) **	0.79 (0.67,0.93) **
Vitamin D supplements	0.59 (0.44,0.80)	0.52 (0.37,0.72) **	0.51 (0.37,0.71) **

*: <0.05, **: <0.001. ^a^ Adjusted for maternal gestational age, pre-pregnancy BMI, mode of conception, maternal ethnicity, gravidity, parity, maternal education level, drinking during pregnancy, smoking during pregnancy, exercise during pregnancy, passive smoking during pregnancy, family income, season of birth of infant, parental allergy history. ^b^ Adjusted for all covariates from adjusted Model 1 and additionally adjusted for hypertension during pregnancy, diabetes mellitus during pregnancy, and thyroid disease during pregnancy.

**Table 4:** Stratified analysis of the association between vitamin D supplementation during pregnancy and infant AD (N = 4051).

Vitamin D Supplementation	Unadjusted Model	Adjusted Model 1 ^a^	Adjusted Model 2 ^b^
	RR (95%CI)	RR (95%CI)	RR (95%CI)
First-trimester 25(OH)D < 50.0 nmol/L			
During mid-pregnancy			
None	Ref.	Ref.	Ref.
Only multi-vitamin supplements	0.72 (0.53,0.97) *	0.66 (0.48,0.92) *	0.64 (0.46,0.89) *
Vitamin D supplements	0.44 (0.26,0.74) *	0.40 (0.23,0.71) *	0.38 (0.22,0.68) *
During late pregnancy			
None	Ref.	Ref.	Ref.
Only multi-vitamin supplements	0.85 (0.64,1.14)	0.79 (0.58,1.08)	0.80 (0.58,1.09)
Vitamin D supplements	0.54 (0.34,0.88) *	0.40 (0.23,0.70) *	0.41 (0.23,0.71) *
First-trimester 25(OH)D ≥50.0 nmol/L			
During mid-pregnancy			
None	Ref.	Ref.	Ref.
Only multi-vitamin supplements	1.01 (0.81,1.26)	0.94 (0.76,1.16)	0.89 (0.72,1.11)
Vitamin D supplements	1.19 (0.84,1.68)	1.14 (0.81,1.61)	1.10 (0.77,1.56)
During late pregnancy			
None	Ref.	Ref.	Ref.
Only multi-vitamin supplements	0.90 (0.74,1.11)	0.80 (0.66,0.99) *	0.80 (0.65,0.98) *
Vitamin D supplements	0.65 (0.44,0.96) *	0.63 (0.42,0.94) *	0.62 (0.41,0.94) *

*: <0.05. ^a^ Adjusted for maternal gestational age, pre-pregnancy BMI, mode of conception, maternal ethnicity, gravidity, parity, maternal education level, drinking during pregnancy, smoking during pregnancy, exercise during pregnancy, passive smoking during pregnancy, family income, season of birth of infant, parental allergy history. ^b^ Adjusted for all covariates from adjusted Model 1 and additionally adjusted for hypertension during pregnancy, diabetes mellitus during pregnancy, and thyroid disease during pregnancy.

## Data Availability

The data described in the manuscript, code book, and analytic code will be made available upon reasonable request to the corresponding authors.

## Supplementary Materials

The following supporting information can be downloaded at: https://www.mdpi.com/article/10.3390/nu16132168/s1, Figure S1: Subgroup analysis of maternal first-trimester vitamin D levels in early pregnancy and the risk of AD in infants; Table S1: Association between vitamin D supplementation during pregnancy and infant AD, stratified by 25(OH)D in early pregnancy (N = 4051).

## Abbreviations

AD, atopic dermatitis; ART, assisted reproductive technology; BMI, body mass index; IPTW, inverse probability weighting; OWO, overweight or obese; PS, propensity score; RR, relative risk; SD, standard deviation.
