# Supplementary material for: Correlation of Maternal Vitamin D Status in Early Pregnancy and Vitamin D Supplementation during Pregnancy with Atopic Dermatitis in Infants: A Prospective Birth Cohort Study
Source: Nutrients. 2024 Jul 8;16(13):2168. doi: 10.3390/nu16132168 (PMC11243106; doi:10.3390/nu16132168)
Supplement: Supplementary file 1 [file nutrients-16-02168-s001.zip › nutrients-3077095-supplementary.pdf]

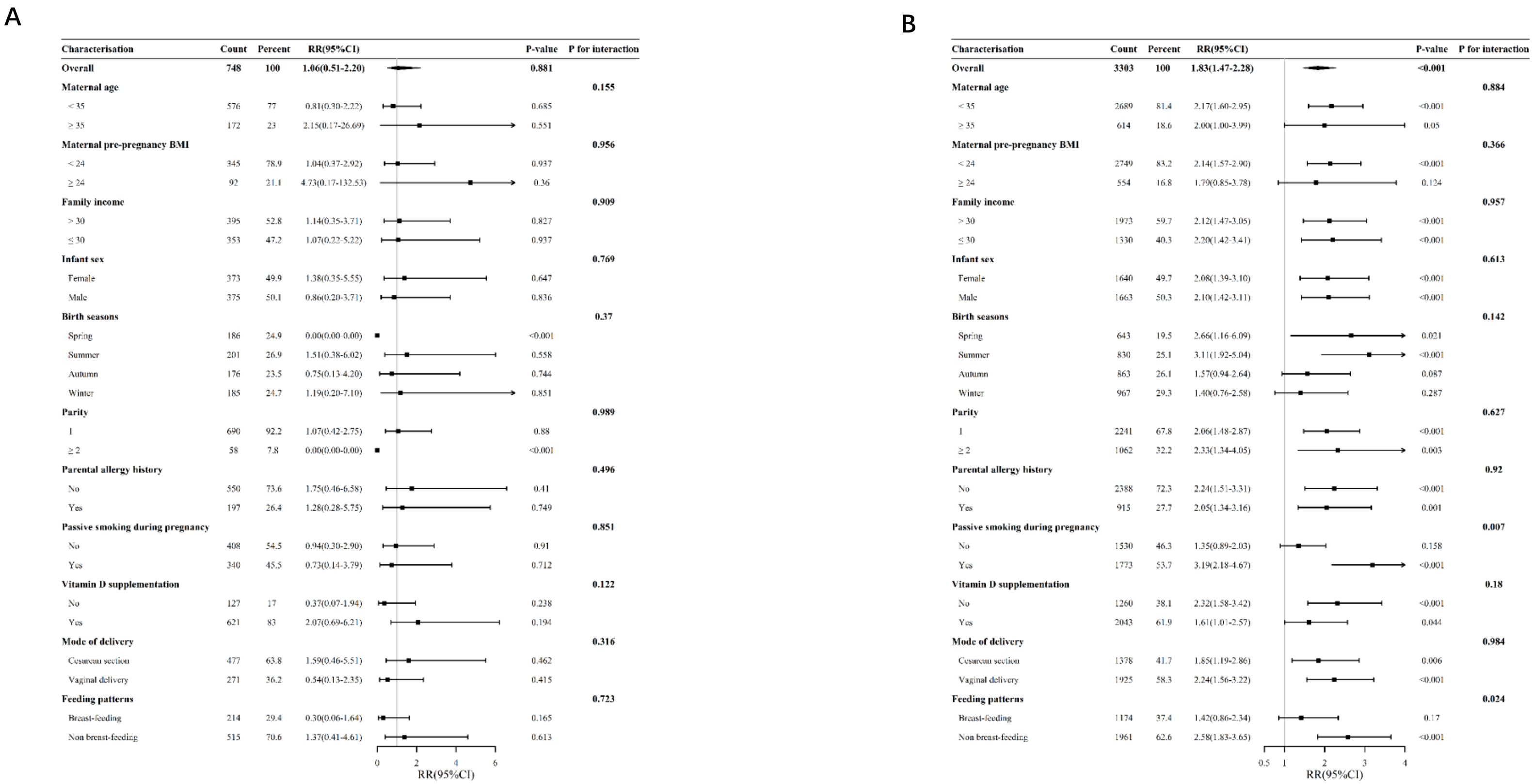

Supplementary Figure S1. Subgroup analysis of maternal first-trimester vitamin D levels in early pregnancy and the risk of AD in infants. Forest plot of the association between serum vitamin D deficiency and risk of infant AD in mothers who conceived through ART (A), and mothers who conceived naturally (B). The subgroup analyses were conducted according to the variable factors and presented in forest plots according to whether maternal first-trimester serum 25(OH)D was deficient (<25 nmol/L), or not (≥25 nmol/L). The analyses were adjusted for all covariates from adjusted model 1, which included maternal gestational age, pre-pregnancy BMI, mode of conception, maternal ethnicity, gravidity, parity, maternal education level, drinking during pregnancy, smoking during pregnancy, exercise during pregnancy, passive smoking during pregnancy, family income, season of birth of infant, parental allergy history, hypertension during pregnancy, diabetes mellitus during pregnancy and thyroid disease during pregnancy. Stratification variables were not included in the adjustment.

Supplementary Table S1. Association between vitamin D supplementation during pregnancy and infant AD, stratified by 25(OH)D in early pregnancy (N = 4051)

Vitamin D supplementation	Unadjusted model	Adjusted model 1 [a]	Adjusted model 2 [b]
	RR (95%CI)	RR (95%CI)	RR (95%CI)
Vitamin D supplementation			
None	Ref.	Ref.	Ref.
In mid-pregnancy	0.88(0.68,1.15)	0.91(0.7,1.18)	0.87(0.67,1.13)
In late pregnancy	0.73(0.52,1.02)	0.73(0.52,1.02)	0.73(0.52,1.03)
In mid- and late pregnancy	**0.79(0.64,0.96)** *	**0.72(0.59,0.88)** *	**0.69(0.56,0.85)** **
**≥ 50.0 nmol/L in early pregnancy**			
Vitamin D supplementation			
None	Ref.	Ref.	Ref.
In mid pregnancy	1.00(0.73,1.37)	0.98(0.71,1.34)	0.95(0.70,1.29)
In late pregnancy	0.67(0.44,1.02)	0.69(0.45,1.04)	**0.66(0.44,0.99)** *
In mid and late pregnancy	0.89(0.70,1.14)	0.83(0.65,1.07)	**0.77(0.60,0.99)** *
**< 50.0 nmol/L in early pregnancy**			
Vitamin D supplementation			
None	Ref.	Ref.	Ref.
In mid-pregnancy	0.67(0.42,1.09)	0.66(0.40,1.09)	0.68(0.42,1.12)
In late pregnancy	0.87(0.48,1.57)	0.93(0.51,1.69)	0.95(0.51,1.77)
In mid- and late pregnancy	**0.64(0.45,0.91)** *	**0.58(0.40,0.84)** *	**0.57(0.39,0.84)** *

*, $P < 0.05$; **, $P < 0.001$.

[a] Adjusted for maternal gestational age, pre-pregnancy BMI, mode of conception, maternal ethnicity, gravidity, parity, maternal education level, drinking during pregnancy, smoking during pregnancy, exercise during pregnancy, passive smoking during pregnancy, family income, season of birth of infant, parental allergy history;

[b] Adjusted for all covariates from adjusted model 1 and additionally adjusted for hypertension during pregnancy, diabetes mellitus during pregnancy, and thyroid disease during pregnancy
